# Evaluation of dose‐area product of common radiographic examinations towards establishing a preliminary diagnostic reference levels (PDRLs) in Southwestern Nigeria

**DOI:** 10.1120/jacmp.v17i6.6011

**Published:** 2016-11-08

**Authors:** Nnamdi N. Jibiri, Christopher J. Olowookere

**Affiliations:** ^1^ Department of Physics, Radiation and Health Physics Laboratory University of Ibadan Oyo State Nigeria; ^2^ Department of Physics Ajayi Crowther University Oyo Town Oyo State Nigeria

**Keywords:** dose‐area product, regional diagnostic reference levels, dose optimization

## Abstract

In Nigeria, a large number of radiographic examinations are conducted yearly for various diagnostic purposes. However, most examinations carried out do not have records of doses received by the patients, and the employed exposure parameters used are not documented; therefore, adequate radiation dose management is hindered. The aim of the present study was to estimate the dose‐area product (DAP) of patients examined in Nigeria, and to propose regional reference dose levels for nine common examinations (chest PA, abdomen AP, pelvis AP, lumbar AP, skull AP, leg AP, knee AP, hand AP, and thigh AP) undertaken in Nigeria. Measurement of entrance surface dose (ESD) was carried out using thermoluminescent dosimeter (TLD). Measured ESDS were converted into DAP using the beam area of patients in 12 purposely selected hospitals. Results of the study show that the maximum/minimum ratio ranged from 3 for thigh AP to 57 in abdomen AP. The range of determined mean and 75th percentile DAPs were 0.18–17.16, and 0.25–28.59 Gy cm^2^, respectively. Data available for comparison show that 75th percentile DAPs in this study (in chest PA, abdomen AP, pelvis AP, lumbar AP) are higher than NRPB‐HPE reference values. The DAP in this study is higher by factor of 31.4 (chest PA), 9.9 (abdomen AP), 2.2 (pelvis AP), and 2.1 (lumbar AP) than NRPB‐HPE values. The relative higher dose found in this study shows nonoptimization of practice in Nigeria. It is expected that regular dose auditing and dose optimization implementation in Nigeria would lead to lower DAP value, especially in abdomen AP. The 75th percentile DAP distribution reported in this study could be taken as regional diagnostic reference level in the Southwestern Nigeria; however, a more extensive nationwide dose survey is required to establish national reference dose.

PACS number(s): 87.53.Bn, 87.59.B

## I. INTRODUCTION

In Nigeria, over 2.5 million medical examinations involving conventional radiography and CT examinations are performed annually in the 36 states of the country. The number of examination is on the increase yearly due to the availability of facilities and the swelling number of CT scanners brought to the country either by government or individuals.

Environmental monitoring of radiation levels in Nigeria has been actively carried out since 1964 after a nuclear weapon was reportedly tested in the Sahara Desert which is close to the northern border of the country.^(1)^ Radiation monitoring equipment was mounted on the rooftop of the building that housed the then Federal Radiation Protection Service (FRPS) to monitor the possible nuclear fallout from Sahara Desert test. Earlier attempt at measuring radiation dose imparted to the patients started at the turn of last millennium.^(2)^ The project was sponsored by IAEA. Some other sponsored dose measurement activities were also carried out thereafter in Nigeria.^3^, ^4^, ^5^ All the sponsored works carried out their investigations in few hospitals (3 to 4) and in well‐equipped X‐ray diagnostic centers within the southwestern part of Nigeria. Other few attempts of dose measurements were also made by individuals (sponsored by individuals).^(6,7,8,9,10,11)^

Both occupational and patient doses are included in the measurement carried out, however only a handful of the results of investigation (sponsored researches) got back to the management of the centers investigated. Besides, there are only few published pediatric dose data in the country. The process of recording the technique factors and patient parameters used during examination as required by National Occupational Health and Safety Commission NRPB, UK^(12)^ for future use, and decision‐making is generally not available in Nigeria.

With the expansion in the use of X‐ray imaging during the last 20 years in Nigeria, extensive dose measurement and effective dose management mechanism are required for dose optimization in the country. Other essential ingredients for dose optimization are documentation of exposure parameters used, feedbacks on the dose measured/parameters selected, and education of the individuals responsible for the exposure of patients. Regular assessment of dose delivered to the patient is deemed necessary and required in every diagnostic center during diagnostic examinations,^(12)^ and the dose should be kept as low as reasonably achievable (ALARA). Other important aspects of radiation protection strategy in diagnostic radiology are contained within the field of quality assurance and quality control.^(13)^

Since radiation could lead to immediate and late effects, radiation dose measurement is required, and it is an essential component of quality control (QC) program. It is indicated in the document of ICRP Report 105 that, in the case of exposure from diagnostic and interventional medical procedures, the diagnostic reference levels (DRLs) have the optimization of protection as its objective.^(13)^ Diagnostic reference level is an important tool for radiation dose management. It is a means to an end — optimization of protection. The concept of DRL was introduced as a tool that identifies the practices with abnormally high patient doses. These guidance levels are defined as dose levels for a typical examination for groups of standard‐sized patients or standard phantom for broadly defined types of equipment. These levels are expected not to be exceeded for standard radiographic procedures when good and normal practices regarding diagnostic and technical performance are followed.^14^, ^15^ Reference levels are intended to act as thresholds to trigger investigations or corrective actions in ensuring optimized protection of patients and maintaining appropriate levels of good practice.^(16)^ Further guidance on what action to be taken was given by the Institute of Physics and Engineering in Medicine (IPEM) through the introduction of remedial level and suspension level at which remedial action was necessary and equipment should be removed from clinical use. Specific values of the ESD on the remedial level and the suspension level were suggested by the IPEM.^(17)^

The DRLs can be determined at local, regional, and national levels. The local diagnostic reference level is defined as the values established by organizations.^(18)^ When organizations (such as large hospitals containing several X‐ray units) take it upon themselves to establish and set their own local diagnostic reference levels (LDRLs), they should have the ability to adapt to a local practice and optimize exposure more effectively.^(19)^ The local diagnostic reference levels reflect the local situation within a large hospital and hence allow for more effective control.^(20)^ The LDRL is not the state‐of‐art but rather the state‐of‐practice.^13^, ^16^ The LDRLs are based on the overall mean (of ESD/DAP) of all rooms. It is not expected that the LDRLs should exceed national diagnostic reference levels (NDRLs) if an organization is undertaking good practice.

With the establishment of LDRLs within a geographical region, it is easier to compare the local performance of a particular hospital with the group of hospitals in a geographical setting expressed in terms of regional diagnostic reference levels. A nation‐wide dose measurement can also be carried out on standard‐sized patients to determine national diagnostic reference levels (NDRLs). As a consequence of the fact that dose distribution in diagnostic radiology examinations is usually skewed with long tail at the higher dose end of the scale, it has been recommended that the 75th percentile of the dose distribution is an appropriate level for DRLs.^(21)^ This is an indication that 75% of the dose distribution falls within the acceptable limit, while the remaining 25% needs attention.

In Nigeria, few studies on patient doses were carried out and published in the past; however, there is no systematic and consistent dose data collection on patient exposure. Moreover, diagnostic reference dose in either conventional radiography or computed tomography (CT) is yet to be determined to the best of our knowledge in Nigeria. The aim of this study was to investigate the current levels of patient radiation dose in Nigeria and explore the possibility of proposing preliminary regional diagnostic reference levels.

## II. MATERIALS AND METHODS

Different methods of estimating entrance surface dose (ESD) are suggested in the European Commission Guidelines.^21^, ^22^ One of the methods suggested was adopted in the present study to determine the entrance surface dose. According to European Commission Guidelines, entrance surface dose can be measured with a thermoluminescent dosimeter (TLD) chip placed on the patient body. In this study, calibrated LiF phosphors were used to measure the entrance surface dose of 600 patients undergoing routine diagnostic examinations. The TLD chips were obtained from Stanford Dosimetry LLC, Bellingham, WA, USA. Facilities of National Institute for Radiation Protection and Research (NIRPR), Department of Physics, University of Ibadan, were used to irradiate and calibrate the chips (of dimension 3×3×1mm). The TLD chips were coded batch‐by‐batch for easy identification before irradiation. Each batch consisting of 10 preannealed chips were irradiated using X‐80‐ X225 X‐ray Beam Irradiator (Hopewell Design Inc., Alpharetta, GA). After irradiation, the TLD chips were left for 24 hrs before calibration. During the calibration of the TLD chips, element correction coefficients (ECC) and reader calibration factors (RCF) were calculated using Harsaw TLD Reader Model 4500 and WinRems software (Saint‐Gobain Crystals & Detector, Wermelskirchen, Germany). The calibrated chips were annealed using an oven provided by Thermoluminescent Laboratory of the Center for Energy Research and Development (CERD), Obafemi Awolowo University, Ile Ife. The TLD chips were annealed under the temperature of 400°C for 1 hr and allowed to cool down in the oven for 17 hrs. They were further kept for 24 hrs before use after each annealing. During routine exposure of patients, a pair of highly sensitive and tissue‐equivalent LiF (TLD‐100) coded dosimeters were placed in the primary beam of the X‐rays where the beam intercepted the patient to measure the ESD (mGy). After each exposure, patient information and the exposure parameters were recorded against the identification numbers of the chips for data processing.

The dose‐area products (DAPs) were calculated from the measured ESD (to air without backscatter) as the product of it and the beam area. The dose‐area product (DAP) is more practical since it better expresses the biological effect of radiation dose. Exposure parameters such as potential (kVp), tube load (mAs), focus‐to‐skin distance (FSD), filtration of the machine (inherent and added), exposed film area (A), thickness of the irradiated part of the body, and projections (AP, PA) were recorded during the routine examination. Other patient data such as height, weight, sex, and age of the patients were also recorded at the time of examination. Data were collected during the period between November, 2011 and March, 2014. Only films that were considered suitable for diagnosis by the radiologist were included in the study in all the hospitals. This ensured that all dose levels used were representative of diagnostic image.

The outputs of the machine in mGy(mAs)^‐1^ at a distance of 1 m were measured using calibrated QC kit (NEROTM 6000M, Victoreen, Inc., Cleveland, OH). This was also used to test linearity and reproducibility of kV. The outputs of the machine were measured at a voltage of 80 kV and 10 mAs as the tube loading.^(23)^ The QC kit was cross‐calibrated at the facilities of Secondary Standard Dosimetry Laboratory (SSDL) of the National Institute of Radiation Protection and Research (NIRPR). The cross‐calibration is traceable to the National Institute of Standards and Technology (NIST). The QC kit (NERO 6000M) was used to check the output of 11 out of 15 units investigated to ascertain their reproducibility at 80 kV and 10 mAs. Similar checks were carried out on tube potential to ensure that the value measured was within the acceptable tolerance limit. Data analysis was carried out using Excel 2007 software.

Twelve purposely selected hospitals consisting of 15 X‐ray units in five states of southwestern (SW) geopolitical zones (Lagos, Ogun, Oyo, Osun, Ekiti) of Nigeria in line with the European Commission guidelines^(21)^ were investigated in this study. Different types of hospitals were included in the study: one Federal Medical Centre (Federal Medical Centre (FMC), Ido Ekiti), three Teaching Hospitals (LAUTECH Teaching Hospital (LTH), Osogbo; Obafemi Awolowo Teaching Hospital Annex (OAUTHW), Ilesa; Ekiti State University Teaching Hospital (EKSUTH)), five private hospitals (Victory Hospital (VHS), Iwo; Seventh Day Adventist Hospital (SDAH), Ile‐Ife; Ayotola Hospital (AYHS), Sagamu; Anikilaya Hospital (ANHS), Ijebu Ode; and Two Tees Diagnostic Centre (TTPC)), and three state hospitals (Orile Agege General Hospital (OAGSH), Lagos; Ifako‐Ijaye General Hospital (FKJSH), Lagos State; and Alimosho General Hospital (ALSH)).

Thicknesses of the irradiated regions (chest, skull, and abdomen) of each patient were measured during the routine examinations. However, the measurement of patient thickness using a tape does not take into account the composition of the patient, therefore, a simple estimate of patient average thicknesses (equivalent cylindrical diameter $D_{e}$) (trunk and skull) were made from height (cm) and body mass (in g) data by assuming that patient is a water cylinder of unit density (g cm^‐3^).^24^, ^25^

The equivalent cylinder diameter $D_{e}$ includes both weight and height; it takes the average density into consideration. Consequently this carries some information about the body shape. The transformation of patient weight and height data to patient‐equivalent diameter^(25)^ takes care of variability that depends on patient size. In order to allow comparisons to be made between data of different hospitals and nationalities, the National Radiation Protection Board (NRPB), UK Protocol^(26)^ recommends the selection of mean weight of patients of the population within 70±5kg, and excluding those outside 70±10kg, at least for frequent examinations, so that the average value of the doses will be a good indicator of a typical dose to an average patient. Using this approach could significantly reduce the amount of data that could potentially be collected and, for small sample sizes, the average dose may not be typical due to the variation of size and body composition within the band of weights. It is extremely difficult to collect a sizeable data of standard size patient in Nigeria. To overcome this, DAP normalization factor of NRPB^24^, ^27^ was applied to the doses of all the patients measured. The normalization factor transforms the dose of any patient to the dose of a “reference man”.^(27)^ Effective doses were obtained using OrgDose software.^(28)^

## III. RESULTS

In this study, 600 adult patients undergoing routine diagnostic examinations involving 9 different procedures in 12 health‐care centers (consisting 15 X‐ray units) were examined. Table 1 shows specific features of X‐ray units included in the study. Output (mGy (mAs^)‐1^) of 11 units out of 15 units investigated range between 0.02146 and 0.6102 mGy (mAs)^‐1^. Additionally, filtrations of 13 out of the 15 units recorded as shown in Table 1, ranged from 0.9 to 3.0 mm Al. Only five units satisfied the minimum filtration requirement of a good practice, while the remaining eight fall short of minimum legal requirement of 2.5 mm Al.^(29)^ The distribution of mean, range, median, and 75th percentile of DAP (Gy cm^2^) are presented in Table 2. Maximum/minimum ratio (range factor) of DAP for different types of examinations are also presented in Table 2.

**Table 1:** Specific features of X‐ray units investigated (output and exposure rate measured at distance of 1 m and tube potential of 80 kV)

*Centers*	*Model*	*Date Manufactured/Installed*	*Total Filtration (mm Al)*	*Output (mGy/mAs)*	*Exposure Rate (mGy/s)*
FMC	Ralco	‐/2013	2.0	0.3859	0.5267
EKSUTH	Allenger 40	2012/‐	0.9	0.3892	0.4471
OAGSH	Picker	‐/2013	2.5	‐	‐
FKJSH	Generic	2007/2009	2.0	‐	‐
ALSH 1	Generic	2007/2009	2.0	0.4531	0.4467
ALSH 2	Siemens Mobile MiniX‐ray	2013/2013	2.2	‐	‐
TTPC 1	Allenger 525	2007/‐	0.9	0.2069	0.7633
TTPC 2	Allenger 525	2007/‐	0.9	0.3998	‐
VHS	Acoma Japan	1983/‐	‐	‐	‐
LTH 1	Neo Diagnomax	1982/‐	3.0	0.1825	0.3246
LTH 2	Allenger 40	2009/	1.5	0.2889	2.1083
SDAH	–	2009/2011	2.5	0.1938	0.8538
OAUTHW	Siemens	2007/‐	2.7	0.6102	3.500
ANHS	Ralco	‐/2013	2.2	0.3064	0.1722
AYHS	GEC Medical	1974/‐	3.0	0.02146	0.1158

**Table 2:** Patient information and statistical parameters of DAP (Gy cm^2^) distribution among the hospitals

*Exam Type*	*N*	*Mean Weight (kg)*	*Mean Age (yr)*	*Mean DAP (SEM)*	*Range of DAP (Gy cm* ^*2*^ *)*	*Median DAP (Gy cm* ^*2*^ *)*	*DAP 75* ^*th*^ *Percentile (Gy cm* ^*2*^ *)*	*Max/Min*
Chest PA	306	67 (37–120)	44.2 (17–90)	3.06 (0.19)	1.01–39.38	1.15	3.14	39
Abdo AP	20	68 (40–91)	58.7 (31–80)	17.16 (4.96)	1.21–68.61	7.21	28.59	57
Pelvis AP	35	73 (54–98)	47.9 (19–74)	3.28 (0.47)	1.07–12.51	2.44	4.77	12
Lumbar AP	87	71 (46–105)	51.1 (20–76)	2.72 (0.44)	1.04–24.12	1.40	3.20	23
Skull AP	32	65 (48–77)	42.9 (19–80)	4.53 (0.052)	0.63–31.79	2.31	5.06	51
Leg AP	46	69 (40–99)	39.1 (16–82)	1.14 (0.15)	0.057–3.05	0.93	2.04	54
Knee AP	17	71 (63–87)	57.2 (26–82)	1.53 (0.21)	0.11–3.00	1.89	2.09	27
Hand AP	45	64 (40–100)	42.7 (19–90)	0.92 (0.14)	0.21–2.85	0.43	1.44	14
Thigh AP	12	72 (56–80)	30.6 (28–46)	0.27 (0.008)	0.11–0.31	0.23	0.25	3

**Figure 1:**
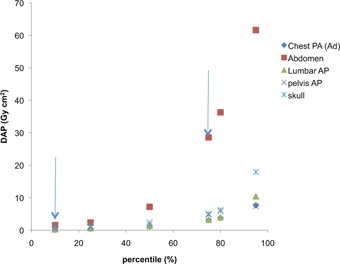
Values of DAP as against the distribution of dose for different procedures (chest, lumbar, pelvis, and skull); arrows indicate 10th and 75th percentiles of the dose distributions.

Figure 1 presents the plot of 10th, 25th, 50th, 75th, 80th, and 95th percentile of the dose distribution for chest PA, lumbar AP, pelvis AP, abdomen AP, and skull AP examinations. The arrow on the right indicates the regional reference dose (75th percentile) determined for the five projections, while the left arrow indicates the action levels (AL) (10th percentile).

The range of mean exposure parameters among different centers are presented in Table 3. A comparison of mean (and range) of tube potential and mAs, with published value used in the UK, USA, and Brazil for each projection, is shown in Table 4. The focus to skin distance and patient‐equivalent diameter for each examination are also shown in Table 4. There were paucity of data on extremities for comparison in UK and Brazil published data. Table 5 shows the effective dose data of male and female patients, body mass index, and equivalent diameter (both obtained from height and weight). Effective doses were obtained using Orgdose software, designed based on ICRP and NRPB data^(28)^ for different examinations.

Dose‐area product (DAP) and effective dose estimated for different procedures are shown in Table 6. Table 7 is a comparison of mean DAP, 75th percentile (diagnostic reference levels) in this study, with NDRLs established in UK and Iran and mean DAP from an earlier study in Nigeria.

**Table 3:** Range of mean exposure parameter among different health–care centers investigated

		*Range of Mean Exposure Parameters Among Health‐care Centers*		
*Exam*	*No. of Centers (n)*	*Tube potential (kVp)*	*Tube load (mAs)*	*Focus to surface distance (FSD in cm)*
Chest PA	15	62–97	8–51	84–173
Abdomen AP	6	78–110	26–100	69–91
Pelvis AP	7	65–87	18–64	81–111
Lumbar AP	12	65–107	23–117	64–122
Skull AP	8	69–88	15–78	64–137
Leg AP	8	48–102	4–80	60–160
Knee AP	12	49–67	5–32	71–108
Hand AP	6	50–53	4–30	48–97
Thigh AP	3	56–78	10–50	82–124

**Table 4:** Comparison of average exposure factors and mAs setting with others used elsewhere (adult)

*Exam Type*	*Tube Voltage (kVp)*	*Tube Load (mAs)*	*FSD (cm)*	*Average Equivalent Diameter (D*_*e*_ *( in cm)*	*NRPB (HPA) UK* ^a^		*USA (N. America)* ^b^		*Brazil (S. America)* ^c^	
					*kVp*	*mAs*	*kVp*	*mAs*	*kVp*	*mAs*
Chest PA	75 (55–185)	25.9 (0.33–100)	125.3 (42–185)	22.4 (16.8–33.9)	88	5	120	–	77	12
Abdomen AP	86 (75–117)	58.1 (20–125)	82.3 (68–99)	22.4 (17.2–25.4)	76	41	75	–	–	–
Pelvis AP	78 (52–92)	35.7 (5–75)	91.9 (63–137)	23.3 (19.9–26.9)	75	33	75	–	–	–
Lumbar AP	85 (60–117)	62.8 (9–200)	76.8 (40–160)	23.2 (19.3–28.7)	78	46	75	10.3	70	84
Skull AP	75 (60–85)	44.4 (8–125)	77.6 (56–122)	22.0 (19.7–24.4)	72	20	80	–	66	59
Leg AP	62 (48–102)	18.7 (3.2–80)	82.4 (60–160)	12.3^d^ (5–33)	–	–	70	10	–	–
Knee AP	63 (58–80)	9.2 (5–32)	87.8 (65–108)	13.5^d^ (9–20)	–	–	70	12	–	–
Hand AP	57 (40–94)	9.2 (2.5–32)	78.8 (52–107)	8.6^d^ (3–18)	–	–	60	4	–	–
Thigh AP	66 (56–75)	17.4 (10–25)	103.3 (82–124)	23.1 (21.5–23.6)	–	–	–	–	–	–

a Hart et al.

b Gkanatsios and Huda.

c Freitas and Yoshimura.

d Measured with a tape.

**Table 5:** Effective doses (mSv) estimated from DAP (Gy cm^2^) for various procedures and patient sizes of male and female adults

*Exam Type*	*N (sex)*	*Effective Dose (mSv)*	*BMI (kg/m* ^*2*^ *)*	*Equiv. Diameter D*_*e*_ *(cm)*
Chest PA	156 (m)	0.44 (0.0052–5.34)	22.85	22.16
	150 (f)	0.39 (0.0012–0.41)	25.66	22.71
Abdo AP	10 (m)	0.89 (0.14–2.93)	23.23	21.74
	10 (f)	2.56 (0.18–7.28)	24.18	23.15
Pelvis AP	21 (m)	0.78 (0.078– 2.20)	23.25	22.56
	14 (f)	0.20 (0.12–0.39)	29.71	24.68
Lumbar AP	48 (m)	0.84 (0.0087–5.86)	24.33	22.28
	39 (f)	0.46 (0.0015–1.69)	26.36	23.09
Skull AP	21 (m)	0.16 (0.021–0.96)	21.77	21.81
	11 (f)	0.078 (0.019–0.24)	21.91	22.55
	30 (m)	—	25.07	11.42^a^
Leg AP	16 (f)	—	28.42	13.92^a^
	35 (m)	—	21.67	8.87^a^
Hand AP	10 (f)	—	19.75	6.21^a^

a Thickness of irradiated part measured with ruler, and the corresponding BMI calculated from height and weight. Abdo AP=abdomen AP

**Table 6:** Dose–area product, DAP (Gy cm^2^), and the equivalent effective doses (mSv) estimated for different procedures for all patients (both male and female)

*Exam*	*DAP (Gy cm* ^*2*^ *)*	*Effective Dose (mSv) (according to ICRP 103)*
Chest PA	3.06	0.42
Abdomen AP	17.16	1.82
Pelvis AP	3.28	0.52
Lumbar AP	2.72	0.66
Skull AP	4.53	0.14
Thigh AP	0.27	0.020

**Table 7:** Comparison of DAP (Gy cm^2^) in this study with values determined elsewhere (NRPB–HPA, Nigeria, and Iran)

	*DAP (Gy cm* ^*2*^ *)*				
*Exam Type*	*This Study (mean)*	*This Study (75* ^*th*^ *percentile)*	*UK (NRPB‐HPA, 2012) NDRLs*	*Mean DAP from Nigeria 2012* ^a^	*Mean DAP and LDRLs from Iran (2013)* ^b^
Chest PA	3.06	3.14	0.1	1.25	0.22
Abdomen AP	17.16	28.59	2.9	0.56	1.29
Pelvis AP	3.28	4.77	2.2	0.46	1.11
Lumbar AP	2.72	3.20	1.5	–	0.71
Skull AP	4.53	5.06	–	0.34	0.42
Leg AP	1.14	2.04	–	–	–
Hand AP	0.92	1.44	–	–	–

a Akinlade et al.

b Shandiz et al.

## IV. DISCUSSION

The information in column 3 of Table 1 indicates that three (VHS, LTH1, and AYHS) of the facilities investigated are very old, and this could affect the performance of the unit except adequate cares are taken. Filtration of the units recorded range between 0.9 and 3.0 mm Al. Only 5 out of 13 units whose filtrations were recorded satisfied the minimum filtration requirement of at least 2.5 mm Al recommended. However, eight units fall short of the legal requirement of 2.5 mm Al filtration^(29)^ for the machines operating at a peak voltage of 70 kV.^(30)^

The use of filtration below the minimum legal requirement of 2.5 mm Al for peak tube potential values greater than 70 kV,^(30)^ especially the newly installed units, could be one of the factors leading to relatively higher patient doses in this study. About 53% of the units were using filtration below the minimum legal requirement of filtration. The filtrations used in the work of Ogundare et al.^(4)^ (UCH: 2.7, BMSH: 1.5, FMC: 2.5 mm Al), Egbe et al.^(31)^ (GH: 2.5, TH: 2.6 mm Al), Akinlade et al.^(32)^ (UCH: 2.7, OAUTHC: 1.7, TDC: 2.7, NHA: 1.0(+0.1mmCu) mm Al), Olowookere et al.^(9)^ (H1: 3.0, H2: 1.0, H3: 2.0 mm Al), and this study show that Nigerian facilities are largely using relatively low filtration compared with the filtrations reported in the work of Suliman et al.^(33)^ which ranged between 2.5 and 5.0 mm Al. The work of Suliman and Elshiekh^(23)^ indicates that low filtrations were used in two out of three hospitals (Omdurman: 2.3 mm Al and A. Gasim: 2.0 mm Al). Wade et al.^(30)^ also show that most of the X‐ray tubes investigated used relatively higher filtration of between 2.5 and 3.5 mm Al.

The study shows that with the appropriate increase in the filtrations while keeping kV and mAs constant, the dose to the patient can be reduced considerably without impairing the image quality. The dose recorded in this study could probably be reduced by increasing the filtration of the machine used in different hospitals while maintaining quality image. Moreover, old X‐ray units could be replaced because of the effect of ageing on the effective output of the machines.

The calculated maximum/minimum ratio (or range factor (RF)) within the hospital and among all the hospitals in this study is an indication of wide variation in DAP (Table 2). The maximum/minimum ratios range from 3 for thigh AP to 57 for abdomen AP. The dose variation could be attributed to many factors, such as patient weight, exposure factors, focus‐to‐surface distance, film‐screen speed, equipment type, and processing performance.^(34)^

The mean weight of patients used in this study falls within 70±10kg (i.e., 64–72 kg), while the mean age falls within the age bracket of the working class in Nigeria (≈ 31–59 yrs). It is also evident from this study that the most frequently carried out examination in Nigeria is chest PA; it is about 51% of the total population studied.

The dose survey is carried out in this study to ascertain the current dose information on common examinations performed in Nigeria. The data shown in columns 5 and 8 of Table 2 are the mean and 75th percentile DAP obtained in Southwestern Nigeria. These could be considered as a preliminary regional diagnostic reference doses against which future dose (DAP) may be compared. The wide variation recorded is an indication that substantial dose reduction could be achieved without loss of image quality.^(35)^ Owing to the wide variation, imaging staff were encouraged to alter their examination techniques in order to make the dose as low as reasonably achievable without compromising the image quality.

Figure 1 shows the plot of DAP against different percentiles (10th, 25th, 50th, 75th, 80th, and 95th). The left and right arrows indicate the 10th and 75th percentile dose distribution. The determined DRLs (75th percentile) and the use of their values serve as a quality improvement tool,^(27)^ but do not indicate the best practice. The DRLs obtained in this study were compared with the mean value calculated from each hospital investigated and were reported to the management of each health‐care center that participated in the study. The possible reasons for poor performance were identified and appropriate steps for better practices were prescribed. The 75th percentile indicated by the right arrow serves as the trigger level, such that any hospital whose mean dose for a given examination exceeds the DRLs requires investigation into the causes of the excessively high dose. It is expected that mean doses for three‐quarters of the hospitals are below the DRLs and one‐quarter are above.^(24)^ The left arrow shows the 10th percentile of the dose distribution, and any radiation doses that are substantially lower than these values may be associated with poor image quality that could result in inadequate diagnostic information.^(27)^ Radiation doses, such as represented by 10th percentile, may require investigation into adequacy of image quality. The 10th percentile is the appropriate action level (AL) at which to initiate an evaluation of the image quality as recommended by International Atomic Energy Agency.^36^, ^37^

In this study, the dose‐area product (DAP) action levels (AL) for chest PA, pelvis AP, skull AP, lumbar AP, and abdomen AP obtained are: 0.14 Gy cm^2^, 0.50 Gy cm^2^, 0.83 Gy cm^2^, 0.25 Gy cm^2^, 1.21 Gy cm^2^, respectively (Fig. 1). The numerical values of AL for different procedures listed above indicate that, if the mean dose at local institution is less than the 10th percentile for the same procedure in the population used to define reference levels, evaluation of image quality should be performed.^(37)^

The range of mean values of exposure parameters shown in Table 3 indicate varied practice of different centers. This large variation among hospitals could be attributed to the experience of each imaging personnel, performance of each machine, expected image quality by referring physician, and the nature of the film‐processing chemicals.

Moreover, the mean exposure parameters used during the routine diagnostic examination for each procedure are shown in Table 4. A comparison of the exposure parameters used in this study with the NRPB‐HPE, USA, and Brazil shows that the mean mAs used in this study for different projections are greater than those used in UK (NRPB), USA, and Brazil. The few exceptions are found in lumbar AP and skull AP (Brazil‐adult patient). The high doses generally recorded in this study could be attributed to relatively higher mAs used, especially in abdomen AP, pelvis AP, lumbar AP, and skull AP. Although some of the kV used in this study are somewhat comparable with the UK, USA, and Brazil values (with the exception of chest PA), it is evident that less attention is paid to patient dose. Apparently, the major concern of the physicians and the imaging scientist is the image quality. Average equivalent diameter of adult patient for the skull and trunk (chest, abdomen, pelvis, and thigh) are in the neighborhoods of value for the standard patient (22.9 cm), that is, in the range of 22.0 and 23.7 cm. The data on extremities from NRPB‐HPE and Brazil are largely unavailable for comparison. The thicknesses of extremities considered in this study were measured with tapes.

Table 5 shows the effective dose distribution, body mass index (BMI), and equivalent diameter ($D_{e}$) of male and female patients. Data for thigh are missing because of few data points. However, Table 5 demonstrates variation in effective dose between the male and female patients.

Effective dose is useful for comparison of doses from different types of examination in general terms for a reference patient, and assessing changes in the dose for a reference patient during the process of optimization. The effective doses range between 0.020 and 1.82 mSv for thigh AP and abdomen AP (Table 6). Apparently, DAP recorded in skull AP is higher than the value obtained in chest PA, pelvis AP, and lumbar spine AP, however the effective doses for the three procedures are higher than the value of the estimated effective dose of skull AP. The reason for this could be the values of different weighting factors used for different body parts. The result of chest PA (0.42 mSv) is higher than the expected effective dose per chest X‐ray (0.05 mSv) by a factor of 8.5. This trend found in Table 6 shows that the practice of “imaging wisely” and “gently” are essential for dose optimization in Nigeria.

When the upper bound of the range of effective doses shown in Table 5 (column 3) is considered, it is clear that high effective doses are being delivered to the patients. For example in chest PA with upper bound of 5.34 mSv (male), the abdomen AP is 7.28 mSv and the lumbar AP for adult female patients is 5.86 mSv.

Dose‐area product for different radiographic views from the present study is compared with data from previous studies in Table 7. Generally, there are paucity of DAP data on extremities for comparison.

Comparison of DAP in this study are made in Table 7 with NRPB‐HPE (latterly called HPE), Iran and earlier DAP data from Nigeria. Generally, the results of DAP (75th percentile) in this study are higher than DAP determined in UK, Iran, and an earlier study from Nigeria. The proposed regional diagnostic reference levels (RDRLs) in this study for different radiographic views are 3.14, 28.59, 4.77, 3.20, 5.06, 2.04, 2.09, 1.44, and 0.25 Gy cm^2^ for chest PA, abdomen AP, pelvis AP, lumbar AP, skull AP, leg AP, knee AP, hand AP, and thigh AP, respectively, for adult patients. The proposed RDRLs in this study are higher than those of NRPB‐HPE by factors which range between 2 and 31, and 3–17 for Iran,^(38)^ respectively. It is no wonder that DRLs observed in this study are greater than the earlier study carried out in Nigeria.^(32)^ The data in the present study were collected in line with the European Commission guidance document.^(21)^ Besides, the doses in this study are normalized to standard patient dose.

The high dose observed in abdomen AP could be attributed to high mAs used during the diagnostic examinations. These relatively high DRLs demand that auditing of the exposure parameters used by different hospital be examined and adjusted. This present study is a first attempt at undertaking a relatively extensive dose measurement in Nigeria towards determining RDRLs. Obviously, this is preliminary and it is essential that more extensive study be carried out nationwide to determine national diagnostic reference levels (NDRLs). A dose database is a necessity and this is the responsibility of Nigerian Nuclear Regulatory Authority (NNRA) in collaboration with National Institute of Radiation Protection and Research (NIRPR) and the National Department of Statistics. A continual dose assessment such as carried out in the United Kingdom (1986, 1995, 2000, 2005, and 2010) and other nations such as the USA and Brazil is necessary in Nigeria to ensure substantial dose reduction in the future. It is evident from this present study that high doses are being delivered to patients during routine diagnostic examinations. It is also clear that patient dose optimization is not yet fulfilled, and diagnostic reference level is not legally adopted in Nigeria. However, it is believed that, through the adoption and regular dose auditing, the current relatively high dose data reported in this study could be reduced to the acceptable levels.

## V. CONCLUSIONS

The results presented in this study provided the current state of practice of radiographic examinations in Southwestern Nigeria. In addition, the results provided preliminary regional diagnostic reference levels PRDRLs of some common diagnostic procedures. The proposed action levels (AL) were also reported in the study. The determined PRDRLs in this study were largely higher than the earlier reference dose determined in UK and Iran. The study is still preliminary; therefore, further extensive dose surveys are required in order to establish a National Reference Dose in Nigeria.

## ACKNOWLEDGMENTS

Authors would like to show their appreciation to the management and staff (Radiologists and Radiographers) of the hospitals and diagnostic centers that participated in the study. Thanks to Professors F.A. Balogun, M.K. Fasasi, and Dr Caleb Aborishade of the Center for Energy Research and Development (CERD) Obafemi Awolowo University, Ile –Ife for making available their TLD facilities for the work. Special thanks go to the staff and management of the National Institute of Radiation Protection and Research (an arm of Nigerian Nuclear Regulatory Authority), University of Ibadan, for assisting us in the calibration and reading of TLD chips. We also appreciate Drs E.K.Osei and R. Barnett of the Department of Medical Physics, Grand River Regional Cancer Center, and Kitchener, ON, Canada for making available OrgDose for this study. Authors also wish to thank the management and staff of Stanford Dosimetry, Bellingham, USA, for supplying the TLD chips used.

## COPYRIGHT

This work is licensed under a Creative Commons Attribution 3.0 Unported License.

## Supporting information

Supplementary Material

## References

[1] Agu BNC . National regulations on the use of radioactive materials. Proc. Workshop Radiation Safety in Nigerian Petroleum Industry: Lagos, Nigeria 24–25 June, 1993.

[2] Ajayi IR and Akinwumiju A . Measurement of entrance skin doses to patients in four common diagnostic examinations by thermoluminescence dosimetry in Nigeria. Radiat Prot Dosimetry. 2000;87(3):217–20.

[3] Ogunseyinde AO , Adeniran SAM , Obed RI , Akinlade BI , Ogundare FO . Comparison of entrance surface doses of some X‐ray examinations with CEC reference doses. Radiat Prot Dosimetry. 2002;98(2):231–34. 11930883 10.1093/oxfordjournals.rpd.a006715

[4] Ogundare FO , Uche CZ , Balogun FA . Radiological parameters and radiation doses of patients undergoing abdomen, pelvis and lumbar spine x‐ray examinations in three Nigerian hospitals. Br J Radiology. 2004;77(923):934–40. 10.1259/bjr/55841517 15507418

[5] Ogundare FO , Ajibola CL , Balogun FA . Survey of radiological techniques and doses of children undergoing some common x‐ray examinations in three hospitals in Nigeria. Med Phys. 2004;31(3):521–24. 15070249 10.1118/1.1644671

[6] Ogundare FO and Ademola JA . Scattered doses to different parts of cancer patients during radiotherapy treatment in Nigeria. Radiat Prot Dosimetry. 2002;102(1):71–74. 12212905 10.1093/oxfordjournals.rpd.a006076

[7] Ogundare FO and Balogun FA . Analysis of occupational dose of workers in the dose registry of the Federal Radiation Protection Service in 2000 and 2001. Radiat Prot Dosimetry. 2003;103(1):57–62. 12596990 10.1093/oxfordjournals.rpd.a006116

[8] Ogundare FO and Balogun FA . Whole body doses of occupationally exposed female worker in Nigeria (1999–2000). J Radiol Prot. 2003;23(2):201–08. 12875552 10.1088/0952-4746/23/2/307

[9] Olowookere CJ , Obed RI , Babalola IA , Bello TO . Patient dosimetry during chest, abdomen, skull and neck radiography in SW Nigeria. Radiography. 2011;17(3):245–49.

[10] Obed RI , Ademola AK , Adewoyin K , Okunade OA . Doses to patients in routine X‐rays examination of chest, skull, abdomen, pelvis in nine selected hospitals in Nigeria. Res J Med Sci. 2007;1(4):209–14.

[11] Jibiri NN and Oguntade GT . Genetically significant dose assessments of occupationally exposed individuals involved in industrial and medical radiographic procedure in certain establishment in Nigeria. Nucl Tech Radiat Prot. 2007;22(2):53–57.

[12] NOHSC . Recommendation for limiting exposure to ionizing radiation. National Occupational Health and Safety Commission (NOHSC). Radiological Protection publication 26. Oxford, UK: Pergamon; 1995.

[13] Charnock P , Moores BM , Wilde R . Establishing local and regional DRLs by means of electronic radiographical X‐ray examination records. Radiat Prot Dosimetry. 2013;157(1). 10.1093/rpd/nct125 23651655

[14] ICRP . Radiological protection in medicine. ICRP Publication105. Ann ICRP. 2007;37(6).

[15] European Community . Council Directive 97/43: Euratom on health protection of individual against the danger of ionizing radiation in relation to medical exposure. L180 40. Luxembourg: European Community; 1997.

[16] Tung CJ , Tsai HY , Lo SH , Guan CN , Chen YB . Determination of guidance levels of dose for diagnostic radiography in Taiwan. Phys Med. 2001;28(5):851–57. 10.1118/1.1368126 11393481

[17] IPEM . Recommended standards for the routine performance testing of diagnostic X‐ray imaging systems. IPEM Report 77. York, UK: Institute of Physics and Engineering in Medicine (IPEM); 1997.

[18] IPEM . Guidance on the establishment and use of diagnostic reference levels for medical X‐ rays examinations. IPEM Report 88. York, UK: Institute of Physics and Engineering in Medicine (IPEM); 2004.

[19] Hadnadjev DR , Arandjic DD , Stojanovic SS , Ciraj‐Bjelac OF , Bozovic Predrag M , Stankovic Jelena S . Patient doses in computed tomography: an assessment of local diagnostic reference levels in large teaching hospital. Nucl Technol Radiat Prot. 2012;27(3):305–10.

[20] The ionizing radiation (medical exposure) regulations 2000. Statutory Instruments No. 1059. London: HMSO; 2000.

[21] EC . Guidance on diagnostic reference levels (DRLs) for medical exposure. Radiation Protection 109. Brussels: The European Commission; 1999.

[22] EC . European guidelines on quality criteria for diagnostic radiographic images. EUR 16260EN. Brussels: The European Commission; 1996.

[23] Suliman II and Elshiekh GH . Radiation doses from some common pediatric X‐ray examinations in Sudan. Radiat Prot Dosimetry. 2008;132(1):64–72. 18765402 10.1093/rpd/ncn232

[24] Hart D , Wall BF , Shrimpton PC , Dance DR . The establishment of reference doses in pediatric radiology as a function of patient size. Radiat Prot Dosimetry. 2000;90(1–2):235–38.

[25] Lindskoug BA . The Reference Man in diagnostic radiology. Br J Radiol. 1992;65(773):431–37. 1611424 10.1259/0007-1285-65-773-431

[26] Dosimetry Working Party of the Institute of Physical Sciences in Medicine . National protocol for patient dose measurement in diagnostic radiology. Clilton, UK: National Radiological Protection Board (NRPB); 1992.

[27] Miller DL , Kwon D , Bonavia GH . Reference levels for patient radiation doses in interventional radiology: proposed initial value in US practice. Radiology. 2009;253(3):753–64. 19789226 10.1148/radiol.2533090354 PMC2786193

[28] Osei EK and Barnett R . Software for estimation of organ equivalent and effective dose from diagnostic radiology procedures. J Radiol Prot. 2009;29(3):361–76. 19690363 10.1088/0952-4746/29/3/001

[29] Johnston DA and Brennan PC . Reference dose levels for patient undergoing common diagnostic X‐ray examination in Irish hospitals. Br J Radiology. 2000;73(868):396–402. 10.1259/bjr.73.868.10844865 10844865

[30] Wade JP , Goldstone KE , Dendy PP . Measurement and dose reduction in East Anglia (UK) Radiat Prot Dosimetry. 1995;57(1–4):445–48.

[31] Egbe NO , Chiaghanam NO , Azogor WE , Inyang SO . A baseline study of entrance dose and image quality for lumbar spine radiography in Calabar, Nigeria. Radiography. 2009;15(4):306–12.

[32] Akinlade BI , Farai IP , Okunade AA . Survey of dose area product received by patients undergoing common radiological examinations in four centers in Nigeria. J Appl Clin Med Phys. 2012;13(4):1–9. 10.1120/jacmp.v13i4.3712 PMC5716517 22766942

[33] Suliman II , Abbas N , Habbani FI . Entrance surface doses to patient undergoing selected diagnostic X‐ray examinations in Sudan. Radiat Prot Dosimetry. 2007;123(2):209–14. 16973669 10.1093/rpd/ncl137

[34] Forward WHM . Patient dosimetry during chest radiography. Radiat Prot Dosimetry. 1995;57(1–4):441–44.

[35] Schneider K , Kohn MM , Bakowski C , et al. Impact of radiographic imaging criteria on dose and image quality in infants in an EC‐wide survey. Radiat Prot Dosimetry. 1993;49(1–3):73–76.

[36] ICRP . 1990 Recommendation of the International Commission on Radiological Protection. ICRP Publication 60. Ann ICRP. 1991;21(1–3). 2053748

[37] Balter S , Miller DL , Vano E , et al. A pilot study exploring the possibility of establishing guidance levels in x‐rays directed interventional procedures. Med Phys. 2008;35(2):673–80. 18383689 10.1118/1.2829868

[38] Shirin Shandiz M , Bahreyni Toosi MT , Farsi S , Yaghobi K . Local reference dose evaluation in conventional radiography examinations in Iran. J Appl Clin Med Phys. 2014;15(2):303–10. 10.1120/jacmp.v15i2.4550 PMC5875487 24710442

