# Supplementary material for: Evaluation of dose‐area product of common radiographic examinations towards establishing a preliminary diagnostic reference levels (PDRLs) in Southwestern Nigeria
Source: J Appl Clin Med Phys. 2016 Nov 8;17(6):392–404. doi: 10.1120/jacmp.v17i6.6011 (PMC5690520; doi:10.1120/jacmp.v17i6.6011)
Supplement: Supplementary file 1 — Supplementary Material [file ACM2-17-392-s001.doc]

**RESPONSE AND OTHER CORRECTIONS**

**Abstract**

Line6 reads…. **therefore,** **this hinders adequate radiation dose management.** …. should read: **therefore, adequate radiation dose management is hindered**.

Line 13 the unit of DAP for the range should be 0.25 – 28.59 **cm^2^ ………**instead of cm2

Line 19 … delete comma between Southwestern and Nigeria … it should be **Southwestern Nigeria**

**I Introduction**

Line 78…reads …..the remaining 25% **need** attention. …. Should read …. the remaining 25% **needs** attention.

**II Materials and Methods**

Line 99……. there should be a space between 17 and hour.. i.e. **17 hours**

Line 123…. a phrase should be added to……. **European Commission guidelines ^(21)^**. To a complete sensible sentence that reads…. **European Commission guidelines ^(21 )^ were investigated in this study.**

Line 131…….. the parenthesis… (**chest, head and abdomen**)….. should read……… (**chest, skull and abdomen**)

Line 134…… the parenthesis …. (trunk and head) should read …..(trunk and skull)

**III Results**

Line 161…… Period before the second sentence should be removed… **Table 3. .A Comparison** … should be **Table 3. A comparison**.

**Lines 168, 169, 170 and 171 …….read …. “Dose area product (DAP) for different projections and its equivalent number of chest x-rays (based on 0.05 mSv per year) and equivalent duration of exposure to nuclear radiation based on 3.0 mSv/year are shown in Table 6.Table 7 is a comparison of mean DAP, 75^th^ percentile (diagnostic reference levels) this present study with NDRLs established in UK, Iran and mean DAP from Nigeria”

Now recast as . .. **Dose-area product (DAP) and equivalent effective dose estimated for different procedures are shown in Table 6. Table 7 is a comparison of mean DAP, 75^th^ percentile (diagnostic reference levels) in this study with NDRLs established in UK, Iran and mean DAP from Nigeria.**

**IV Discussion**

Line 176….. the parenthesis around ref **(^20)^** should be **^(20)^.**

Line 183.. cited ref .. Olowookere et al., 2010 (is one of the authors) …. Therefore, should read “**Author, 2010. Redacted to preserve anonymity of review process”**

Line 189….increase in the filtrations and keeping kV… should read… increase in the filtrations **while** keeping kV

Line 196 ...... Remove the second period after abdomen AP.

Line 200 ….. should read …….**in Nigeria is chest PA, it is about 51% of the total population studied**

Line 203 ….. **This** becomes… **these,** and reference dose becomes **reference doses**

Line 224 ….should read …. Abdomen **obtained are** (were replaced)

Line 238 … should read …. and Brazil **values** (with **the** exception of chest PA) (s added to value, the included in the parenthesis)

Line 241 … should read ……. in the range of 22.0 and 23.7 **cm** (unit added)

Lines 250 to 257 modified as suggested by the reviewer…. to read (bold type to replace the former)

Line 250…. the process of optimization. **The effective doses range between 0.020 and 1.82 mSv for thigh AP and abdomen AP (Table 6). Apparently, DAP recorded in skull AP is higher than the value obtained in chest PA, pelvis AP and lumbar AP, however, the effective doses for the three procedures are higher than the value of the estimated effective dose of skull AP. The reason for this could be the values of different weighting factors used for different body parts. The result of chest PA (0.42 mSv) is higher than the expected effective dose per chest x-ray (0.05 mSv) by a factor of 8.5**. ……. (Line 257) This trend found in…

Lines 259 – 261 modified to read ….**When the upper bound of the range of effective doses shown in Table 5 (column 3) are considered, it is clear that high effective doses are being delivered to the patients. For example in chest PA with upper bound of 5.34 mSv (male), abdomen AP:7.28 mSv and lumbar AP:5.86 mSv for adult female patients.**

Line 263 …… the second **period** (full stop) at the end of the sentence to be removed.

Line 279 should read ……., and the **National** Department of Statistics (**National** included)

Line 281 should read …….. such as the US, **and** Brazil is **necessary** (**and** included, .. **necessary**  replaced essential)

**References**

Line 302 …… space … **thermoluminesce dosimetry**

Line 314 Reference modified… **radiotherapy treatment in Nigeria. Radiat Prot Dosim. 2002; 102 (1) 71- 4**.

Line 319 ….. Ref 9 .. “**Author, 2010. Redacted to preserve anonymity of review process”**

Line 324…. Ref 11 “**Author, 2011. Redacted to preserve anonymity of review process”**

Line 327 ….. semi colon to be removed

Line 358 Ref 24 paediatric to read **pediatric**

Line 360…. Ref 26 should be Institute of **Physical** Sciences (upper case for P of **Physical**)

Line 269 …..Ref 29 close the gap…… **396 – 402**

Line 381…. Ref 35…. Bakowski C,.. (include comma)

**Line 410 ….. 415 … Table 1 caption should be ….**Table 1: Specific features of x-ray units investigated (output and exposure rate measured at a distance of 1 m and at tube potential of 80 kV)** (this is in line with the suggestions of  **Reviewer D**)

Line 475-479 ….. Table 5 caption should be …….**Table 5: Effective doses calculated from DAP (Gy cm^2^) for different procedures and patient size**

Line 480…… should read.... ***thickness of irradiated parts measured with rule and corresponding BMI calculated from height and weight**

**Line 490….. Table 6 caption should read …. **Table 6:DAP (Gy cm^2^) and equivalent effective doses estimated from DAP for different procedure**

******Table 6 modified as suggested by Reviewer D **…. Columns 4 and 5 of Table 6 deleted**

Line 495 …… Table 6 footnote deleted….(or Line 495 deleted)

** indicates correction carried out in line with the comment of the Reviewer. All the suggestion of Reviewer were adequately addressed and corrected.
